# Surface functionalization dependent subcellular localization of Superparamagnetic nanoparticle in plasma membrane and endosome

**DOI:** 10.1186/s40580-018-0136-3

**Published:** 2018-02-15

**Authors:** Deepak B. Thimiri Govinda Raj, Niamat Ali Khan

**Affiliations:** 1 Envirotransgene® Bio-solutions Global Chennai, Tamil Nadu, India; 2 Biotechnology Centre for Oslo, Centre for Molecular Medicine Norway (NCMM), Blindern, P.O. Box 1137, 0318 Oslo, Norway; 3 Laboratory of Lipid Metabolism and Cancer, O&N I, Herestraat 49, Box 902, 3000 Louvain, Belgium

**Keywords:** Superparamagnetic nanoparticle (SPMNP), Plasma membrane, Early or late endosomes, Lysosomes

## Abstract

In this article, we elaborate the application of thermal decomposition based synthesis of Fe_3_O_4_ superparamagnetic nanoparticle (SPMNP) in subcellular fractionation context. Here, we performed surface functionalization of SPMNP with phospholipids and dimercaptosuccinic acid. Surprisingly, we observed surface functionalization dependent SPMNP localization in subcellular compartments such as plasma membrane, endosomes and lysosomes. By using SPMNP based subcellular localization with pulse–chase methodology, we could use SPMNP for high pure-high yield organelle (plasma membrane, endosomes and lysosome) fractionation. Further, SPMNP that are distinctly localized in subcellular compartments can be used as technology for subcellular fractionation that can complement existing tools for cell biology research. As a future perspective, isolated magnetic organelles can be extended to protein/protein complex purification for biochemical and structural biology studies.

## Introduction

Plasma membrane, endosomes (early or late), and lysosomes are dynamic organelles that are known for their key role in cellular function such as cell signaling [1–3], intracellular [4, 5] and extracellular interaction [6, 7]. Interestingly, most drugs and therapeutic small molecules require cellular interaction for their biological action, and it is well known that high affinity with the cell surface results in effective mechanisms [8]. Several drug candidates for cancer, neurodegenerative and metabolic diseases predominately interact with the proteins at plasma membrane, endosomes and lysosomes [9]. For example, gamma-secretase protein complexes that play a key role in neurodegenerative disease are predominately localized in plasma membrane and endo-lysosomal compartments [10]. Gamma-secretase substrate cleavage activity occurs at the cell surface and endosomal compartments [11]. Hence, it is essential to identify proteins that interact with gamma secretase complex in those subcellular compartments during drug treatment or other perturbations. In order to identify such subtle mechanism, it is essential to characterize the biomolecular composition of plasma membrane, endosomes and lysosomes [12–14]. This is due to the dynamic nature of protein and lipid composition in a cell. For instance, 90% of cholesterol is present at the cell surface in normal cells and it has been observed that majority of the cholesterol is internalized in Presenilin double knockout (PSENdKO) cell lines [15]. Hence, it is essential to isolate organelles such as plasma membrane, endosomes and lysosomes in order to identify subtle changes in protein or lipid composition compared to whole cell [16]. Such an approach will facilitate the analysis of the composition and dynamics of subcellular compartments upon drug exposure. Major limiting factor in determining the biomolecular composition of these dynamic organelles are the difficulties in isolating these organelles with high purity and high yield [17]. There are several existing methodologies such as density gradient centrifugation [18], antibody based immuno-precipitation [19], cationic silica based fractionation [20, 21] and streptavidin based magnetic fractionation [22, 23] that are used to isolate organelles such as plasma membrane with varied purity and yield. Similarly, nanoparticle based fractionation [24]; density gradient centrifugation [25] and antibody based immunoprecipitation [26] are used to isolate dynamic organelles such as endosome and lysosomes with limited purity. However, biomolecular composition of plasma membrane, endosomes and lysosomes are still incomplete for several cell types [27]. SPMNPs based subcellular fractionation is an effective and simple methodology for isolating plasma membrane, early endosomes, late endosomes and lysosomes in any given eukaryotic cell [28]. SPMNP based subcellular fractionation is considered to be superior as organelles are isolated under native physiological conditions devoid of detergents or acidic condition [29]. In addition, SPMNP based subcellular fractionation are generic and can be applied to any living eukaryotic cell [30]. However, synthesis and surface functionalization of SPMNP plays key role in its application. In this article, we show that thermal decomposition synthesis based SPMNP can be localized in two different subcellular compartments using two types of surface functionalization. We particularly show that phospholipid functionalized SPMNPs and dimercaptosuccinic acid (DMSA) functionalized SPMNPs can be used for plasma membrane and endosomal enrichment respectively. In addition, we present methodologies for thermal decomposition based SPMNP synthesis, surface functionalization of SPMNPs, bio-conjugation of SPMNPs with fluorescent phospholipid or by fluorescein-5-maleimide, pulse–chase paradigm and magnetic fractionation for subcellular organelles (plasma membrane, endosomes and lysosomes). As a proof of concept, here we focus on use of SPMNPs and its pulse chase dependent subcellular localization for subcellular fractionation in HeLa cells.

## Experimental section

### Thermal decomposition based synthesis of SPMNP

Using Sun and Zeng’s protocol [31], Magnetite (Fe_3_O_4_) nanoparticles with size from 3 to 20 nm can be produced using simple organic-phase synthesis. We prepared 6 nm Fe_3_O_4_ SPMNP by mixing 2 mmol of iron (III) acetylacetonate with 10 mmol of 1,2-hexadecanediol (10 mmol), 6 mmol of oleic acid and 6 mmol of oleyl amine in 20 ml of benzyl ether. We then magnetically stirred and heated the mixture to 200 °C for 2 h under N_2_ flow for synthesizing SPMNP seed. Reflux at 300 °C for 1 h resulted in SPMNP seeds growing to 6 nM SPMNP. After cooling to room temperature, we added ethanol and separated dark brown precipitate using magnet. We then dissolved dark brown material in hexane and centrifuged at 1000 rpm for 5 min to remove aggregates. Using phospholipids or DMSA as ligand, water dispersible SPMNP are generated in controlled fashion by retaining monodispersity and superparamagnetic properties.

### Phospholipid based surface functionalization of SPMNP

Based on Dubertret et al protocol [32], we performed phospholipid (Pl) functionalization on SPMNP by ligand addition to generate Pl-SPMNP. Briefly we mixed 1:2 ratio of SPMNP with PEG functionalized Phospholipids in chloroform and vortexed for 4 h. The above reaction mixture was gently mixed and incubated for 3 h with gentle and continuous vortexing. After evaporating the chloroform and drying the particles using nitrogen gas, we dissolved the pellet in water (1 ml) by gentle shaking of the sample. We then centrifuged the above reaction mixture for 10 min at 5000 rpm at 21 °C in order to remove the major aggregates. Finally, we extracted the supernatant in a new 1.5 ml centrifuge tube and centrifuged it for 1 h at 5000 rpm at 21 °C in order to get monodispersity.

### DMSA based surface functionalization of SPMNP

Using Thimiri Govinda Raj et al protocol [29], we performed ligand exchange on oleic acid functionalized SPMNP using DMSA as ligand to generate DMSA-SPMNP. We mixed 1:200 ratio of SPMNP with DMSA in 1:1 toluene and dimethyl sulfoxide (DMSO) solution. Then reaction mixture was vortexed for 48 h at room temperature. After 48 h, the black precipitate was pelleted through centrifugation at 5000 rpm for 10 min. Using glass pipette, the toluene and DMSA solvent mixture was discarded. The pellet was resuspended in appropriate volume of water by vigorous sonication. Finally SPMNP in water was adjusted to pH 7.

### TMAOH and COO-TMACl based surface functionalization of SPMNP

Using Thimiri Govinda Raj et al protocol [29], we performed ligand exchange on oleic acid functionalized SPMNP using TMAOH and COO-TMACl as ligand to generate TMAOH or and COO-TMACl-SPMNP. We mixed 1:200 ratio of SPMNP with TMAOH or COO-TMACl in 1:1 toluene and methanol solution. Then reaction mixture was vortexed for 48 h at room temperature. After 48 h, the black precipitate was pelleted through centrifugation at 5000 rpm for 10 min. Using glass pipette, the toluene solvent mixture was discarded. The pellet was resuspended in appropriate volume of water by vigorous sonication. Finally SPMNP in water was adjusted to pH 7.

### Generation of fluorescent SPMNP

We used two strategies to generate fluorescent SPMNP depending on the ligand functionalization (a) fluorescent Pl-SPMNP: 1:1:4 ratio of oleic acid functionalized SPMNP with PEG functionalized Phospholipids and fluorescent end grouped PEG functionalized Phospholipids respectively in chloroform was mixed. (b) Maleimide linker based conjugation of SPMNP [33]: 1:25 molar ratio of SPMNP with fluorescein-5-maleimide in water at pH 7.0 was mixed. The reaction mixture was incubated in mild shaking for overnight at 4 °C. Using magnetic separation, unconjugated fluorescein-5-maleimide was removed. The magnetically purified fluorescein conjugated SPMNPs was resuspended in water at pH 7.0.

### Subcellular barcoding—choice of SPMNP for pulse–chase paradigm

In order to isolate subcellular organelles, it is critical to select appropriate SPMNP that selectively localize at subcellular compartments. To select SPMNP that are cell surface bound or internalized in cell, magnetic cell isolation protocol with different pulse and chase conditions are to be performed. Adherent mammalian cells (ex: HeLa, MEFs) were incubated with different concentration of SPMNPs in medium at 4 or 37 °C for 15 min as pulse period. The cells were trypsinized and harvested at 1000 rpm for 10 min. Magnetic cell isolation was performed using the protocol for magnetic separation from Miltenyi Biotec.

### Prussian blue staining

Adherent mammalian cells to 75% confluence were cultured in 6 well plates with coverslips. Further cells were incubated at 37 °C with SPMNPs for pulse period. After washing with PBS, cells were incubated at 37 °C in fresh medium devoid of SPMNP for chase period (0 and 240 min). Then cells were washed with ice-cold PBS three times and fixed with paraformaldehyde. Cells were incubated with fresh reagent- potassium ferrocyanide (2%): hydrochloric acid (2%) = 1:1 for 10 min and repeated with the fresh reagent. The cells were then rinsed with distilled water, counter stained with eosin for 30 min and washed again with tap water. The SPMNP (blue staining) localization was captured using light microscope.

### Confocal analysis

Adherent mammalian cells to 75% confluence were cultured in 6 well plates with coverslips. Further cells were incubated at 37 °C with SPMNPs for pulse period. After washing with PBS and cell were incubated cells at 37 °C in fresh medium with lysotracker devoid of SPMNP for chase period (0 and 240 min). Then cells were washed with ice-cold PBS three times and fixed with paraformaldehyde. Cells were rinsed with PBS and SPMNP localization was captured using confocal microscope.

### Western blot analysis on SPMNP based subcellular fractionation

Adherent mammalian cells to 75% confluence were cultured in 75 cm^2^ flask or in 8 × 10 cm culture-dishes at 37 °C and washed three times with warm PBS. The cells were then incubated with SPMNP in medium (0.2 mg/ml) for pulse time of 15 min at 37 °C in a mild shaking platform. To capture plasma membrane, cells with NH_2_-lipid-SPMNP in PBS (2 mg/ml) were incubated for pulse time of 15 min at 4 °C. Excess SPMNPs were removed by washing the cells with PBS, and a chase period of 0–15 h with fresh medium at 37 °C was performed (chase period—0 min to capture plasma membrane, chase period—15 min to 2 h to isolate endosomes and chase period 2–15 h to isolate lysosomes). The cells were harvested in PBS by centrifuging at 1000 rpm for 10 min. The cell pellet was resuspended in homogenizing buffer and homogenized with ball-bearing cell cracker (20 passages, clearance 10 or 15 µm). The cell debris was pelleted by centrifuging at 200*g* for 10 min and the supernatant as post nuclear supernatant fraction (PNS) was retained. The PNS was loaded in a PBS equilibrated LS column in the presence of magnetic field. The non-magnetic unbound fraction was aliquoted for quality control and western blot analysis. The column was washed in the presence of magnetic field with homogenizing buffer for three times. The bound fraction was removed by the magnetic field and using the plunger. The bound fraction was pelleted using ultracentrifugation in 55,000 rpm for 1 h at 4 °C. Western blot analysis was performed to study the magnetic fraction.

## Result and discussion

### Superparamagnetic nanoparticle (SPMNP) synthesis and surface functionalization

**Fig. 1:**
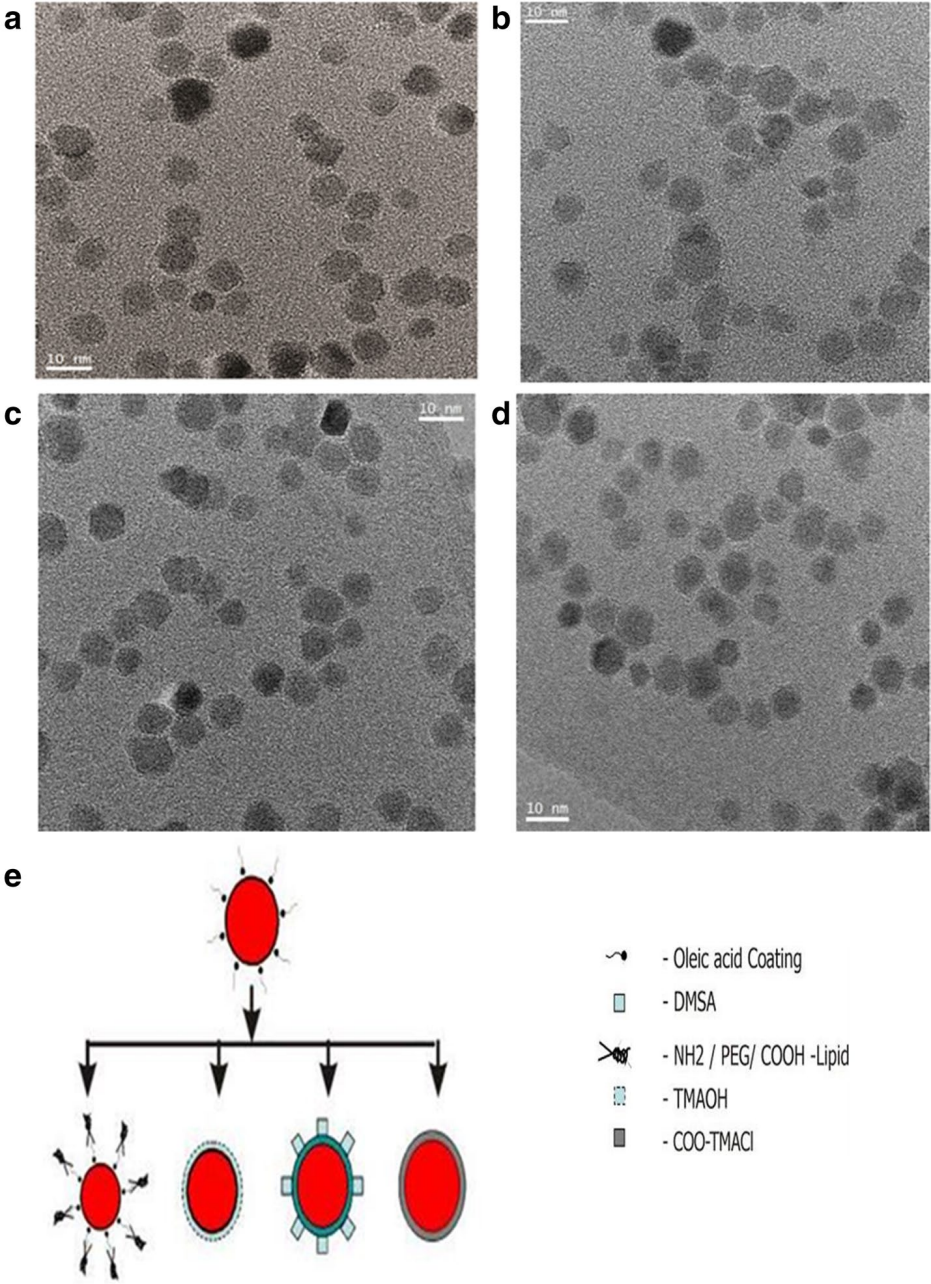
Transmission electron microscopy (TEM) micrograph showing image of (**a**) SPMNP synthesized by thermal decomposition; **b** Lipid coated SPMNP by ligand addition method; **c** DMSA functionalized SPMNP by ligand exchange method; **d** TMAOH functionalized SPMNP and schematic representation of different types of SPMNPs. **e** Schematic diagram for different types of SPMNP synthesis and functionalization

Figure 1 shows the transmission electron microscopy (TEM) images of SPMNP that are synthesized using thermal decomposition (Fig. 1). Synthesized Fe_3_O_4_ nanoparticles are uniform in size with high monodispersity observed for thermal decomposition based SPMNP. Using thermal decomposition based SPMNP as a core nanoparticle, SPMNPs are generated either by ligand addition for lipid-SPMNP or ligand exchange for DMSA-SPMNP, TMAOH-SPMNP are observed to retain monodispersity and core size. Further surface modification by coupling fluorescent molecules (Fluorescein) to SPMNP to generate fluorescent SPMNP doesn’t have major changes in the nanoparticle core properties and monodispersity. Dynamic light scattering (DLS) measurement of oleic acid functionalized SPMNP in hexane shows that nanoparticle size is 11 ± 0.3 nm based on size distribution calculation. DLS measurement of water dispersible SPMNP shows that nanoparticle size is in the range 20 ± 2 to 30 ± 4 nm. In order to apply for SPMNP for biological application, it is necessary that SPMNP are biocompatible and retain physical properties such as size. DLS measurement of DMEM medium dispersed SPMNP shows that Lipid-SPMNP and DMSA-SPMNP retain the hydrodynamic size, while TMAOH-SPMNP and COO-TMACl-SPMNP seem to aggregate with increase in size distribution. In addition, thermal gravimetric analysis (TGA) and Fourier-transform infrared (FTIR) spectra measurements confirm the surface functionalization of SPMNP as previously reported [24]. Hence, TMAOH-SPMNP and COO-TMACl-SPMNP were not used further in this subcellular localization studies. Zeta potential measurement on DMSA-SPMNP, NH_2_-Lipid-SPMNP, COOH-lipid-SPMNP and PEG-Lipid-SPMNP were performed previously confirming negative charge for DMSA-SPMNP, COOH-lipid-SPMNP and PEG-Lipid-SPMNP and positive charge for NH_2_-Lipid-SPMNP [34].

**Fig. 2:**
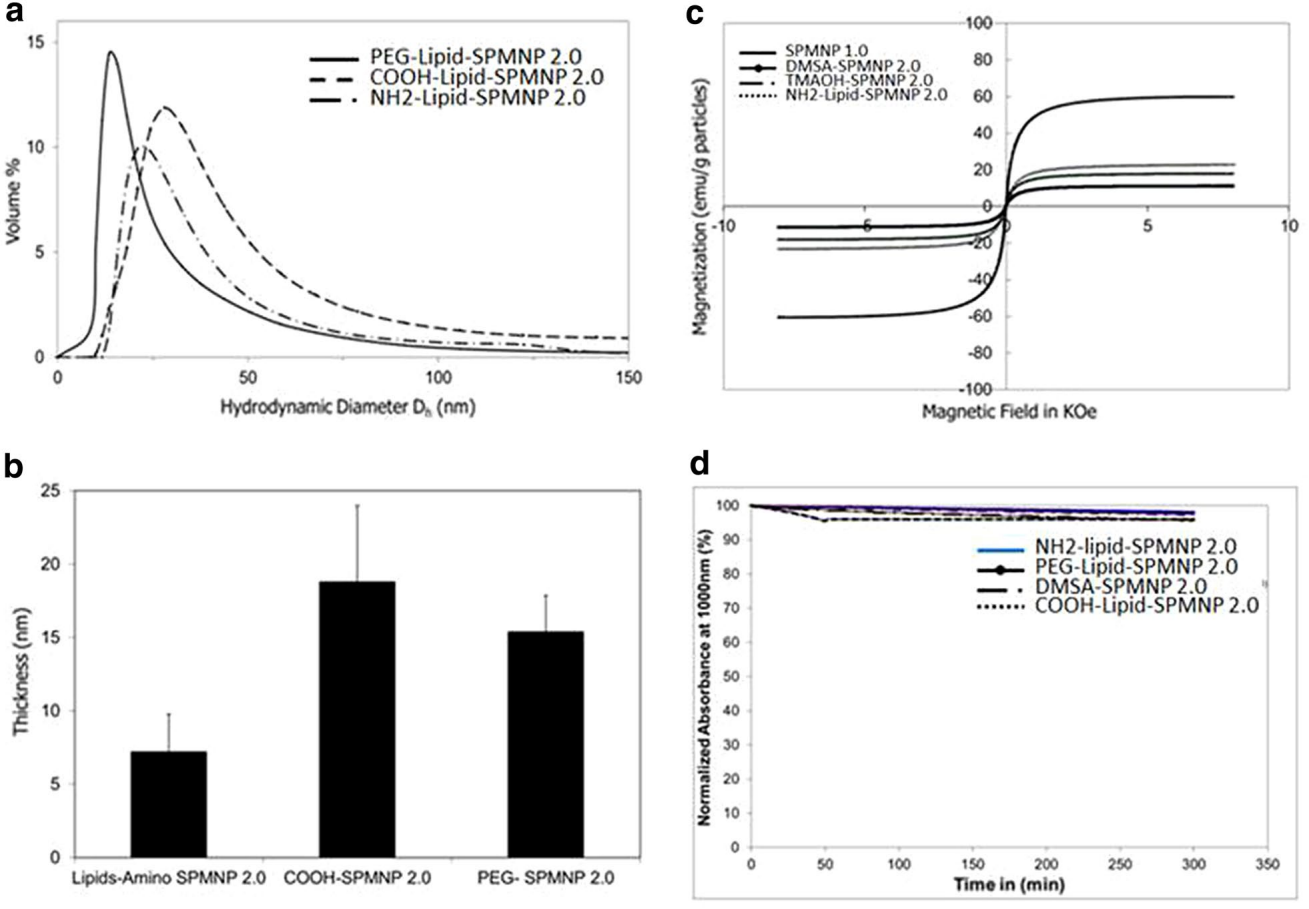
**a** Hydrodynamic diameter D_h_ (nm) based on dynamic light scattering (DLS) micrograph for Lipid-SPMNP by ligand addition method such as NH_2_-lipid, COOH-lipid, and PEG-lipid; **b** hydrodynamic shell thickness of Lipid-SPMNP by ligand addition method such as NH_2_-lipid, COOH-lipid, and PEG-lipid coated SPMNP; **c** magnetization properties (emu/g particles) of SPMNP, DMSA-SPMNP, TMAOH-SPMNP and NH_2_- Lipid-SPMNP; **d** stability of lipid- SPMNP and DMSA-SPMNP in DMEM medium for 5 h

Figure 2 shows differences in hydrodynamic diameters among Lipid-SPMNP such as PEG-endgroup Lipid-SPMNP, NH_2_-endgroup Lipid-SPMNP and COOH-endgroup Lipid-SPMNP that was measured using DLS. Previously Xinlei Huang et al. have reported that shell thickness of Lipid-SPMNP depends on the hydrophobic bilayer and stretching of the PEG tails [35]. Similar observation was inferred with Lipid-SPMNP and confirms charged dependent shell thickness (Fig. 2b). Alternating gradient force magnetometry (AGFM) measurement confirmed superparamagnetic properties of SPMNP DMSA-SPMNP, Lipid-SPMNP and TMAOH-SPMNP (Fig. 2c). Due to additional shell thickness on SPMNP, magnetization emu per g of SPMNP (DMSA-SPMNP: 17.87 emu/g, Lipid-SPMNP: 22.80 emu/g, TMAOH-SPMNP: 11.70 emu/g) is lesser compared to SPMNP (59.60 emu/g) as reported previously [36]. Stability of DMSA-SPMNP and lipid-SPMNP in DMEM medium was analyzed using UV spectrophotometer and inferred to be stable over the period of 5 h (Fig. 2d).

**Fig. 3:**
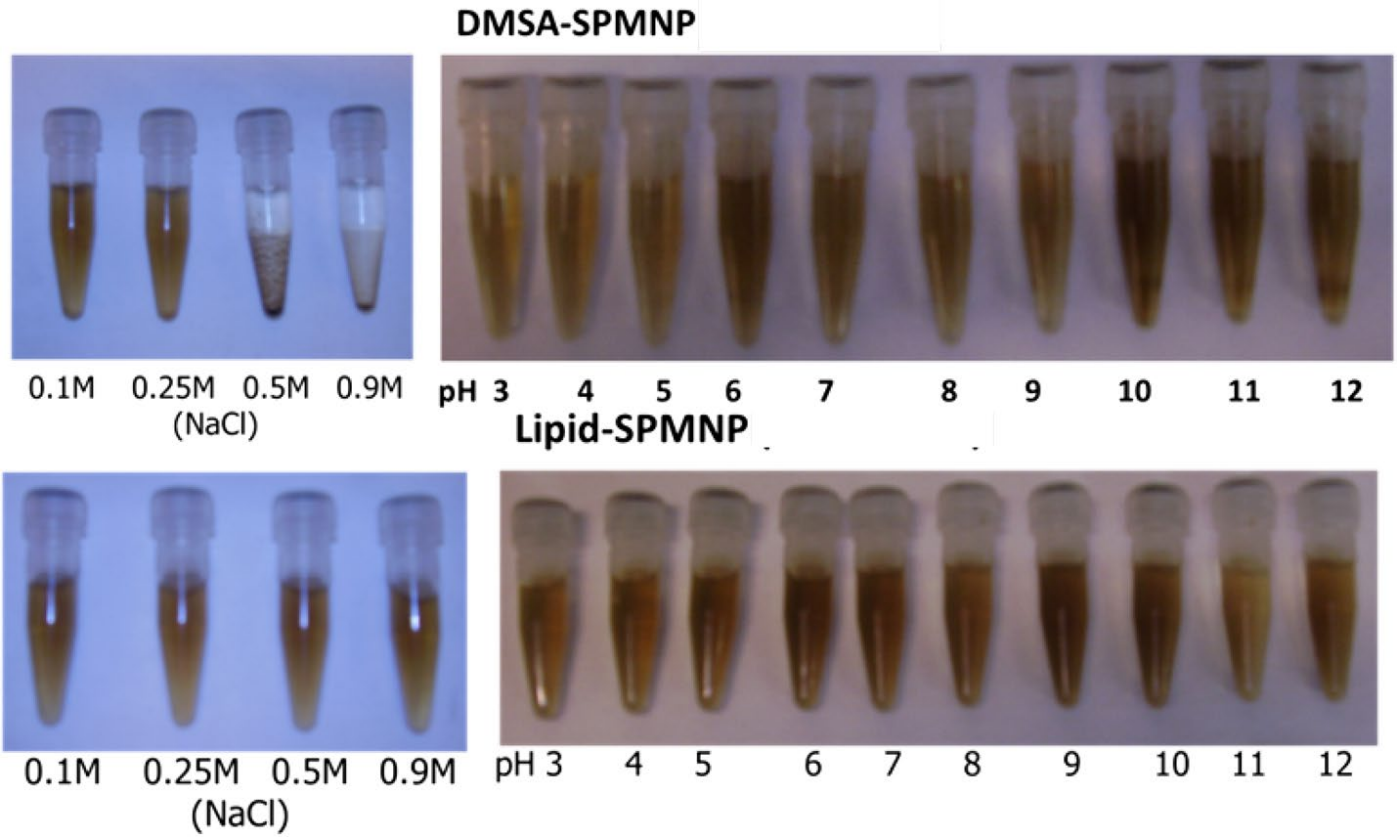
Stability of DMSA-SPMNP and NH_2_-Lipid-SPMNP for increasing NaCl concentration (0.1, 0.25, 0.5, 0.9 M) and increasing pH range (3–12)

Figure 3 shows the stability of DMSA-SPMNP and Lipid-SPMNP for pH range from 3 to 12 and NaCl concentration of 0.1, 0.25, 0.5 and 0.9 M. As reported by Lee et al. [37], DMSA-SPMNP is stable for NaCl concentration of 0.1 and 0.25 M and stable for all pH range. However, Lipid SPMNP is stable for all NaCl concentration (0.1, 0.25, 0.5 and 0.9 M) and the pH range from 3 to 12. The fluorescent DMSA-SPMNP was generated by coupling fluorescein diacetate 5-maleimide with free thiol (-SH) group of DMSA-SPMNP [38].

### SPMNP-cell interaction

**Fig. 4:**
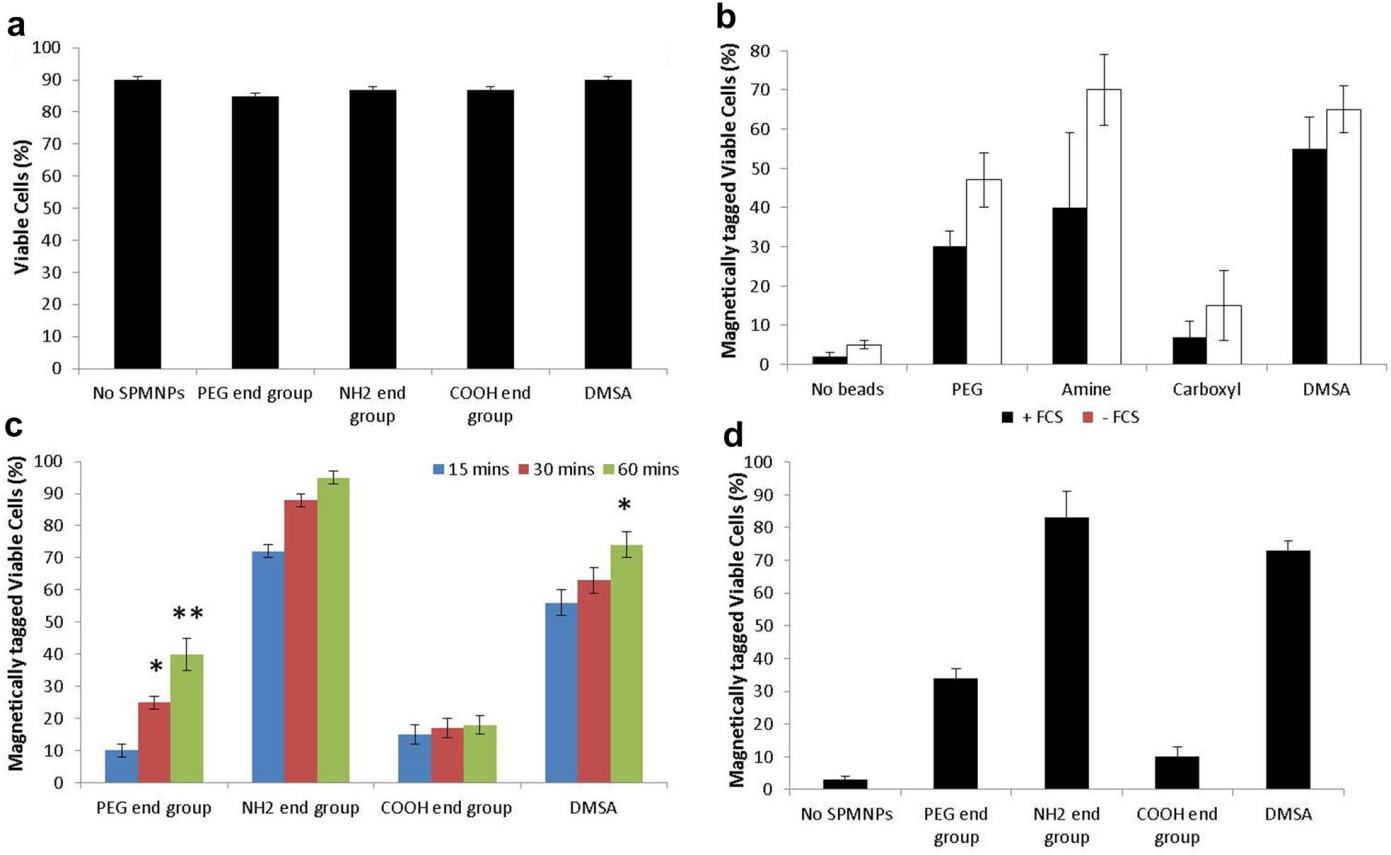
Cell viability and magnetic fractionation: **a** trypan blue staining of HeLa cells incubated with DMEM medium for 2 h with no SPMNP, PEG-Lipid-SPMNP, NH_2_-Lipid-SPMNP, COOH-Lipid-SPMNP and DMSA-SPMNP; **b** magnetically isolated viable HeLa cells incubated with DMEM medium with or without FCS for 2 h with no SPMNP, PEG-Lipid-SPMNP, NH_2_-Lipid-SPMNP, COOH-Lipid-SPMNP and DMSA-SPMNP; **c** magnetically isolated viable HeLa cells incubated with DMEM medium with PEG-Lipid-SPMNP, NH_2_-Lipid-SPMNP, COOH-Lipid-SPMNP and DMSA-SPMNP for pulse period 15, 30 and 60 min; **d** magnetically isolated viable HeLa cells incubated with DMEM medium with PEG-Lipid-SPMNP, NH_2_-Lipid-SPMNP, COOH-Lipid-SPMNP and DMSA-SPMNP for pulse period of 20 min; (T test: *p < 0.5, **p < 0.05)

Figure 4 shows SPMNP interaction with HeLa cell and magnetic tagging of cells for further subcellular fractionation. No cell toxicity was observed with HeLa cells incubated with the SPMNP in DMEM medium (Fig. 4a). Presence of fetal calf serum (FCS) in medium with SPMNP (100 μg/ml) resulted in higher magnetic cell fraction (Fig. 4b). This confirms previous studies that protein corona in the serum determines NP uptake [39]. NH_2_-lipid-SPMNP seems to tag all the cells within 60 min of SPMNP-cell interaction, while DMSA-SPMNP and PEG-lipid-SPMNP shows 70 and 40% magnetic cell tagging respectively (Fig. 4c). COOH-lipid-SPMNP showed no increase in magnetic cell tagging with increase in pulse period. This is due to negative charge of COOH and presence of PEG in lipid layer of COOH-lipid-SPMNP that limits cell surface and protein interaction respectively [40]. Increasing the SPMNP concentration (200 μg/ml) resulted in lesser incubation time of 20 min to magnetically isolate cells using NH_2_-lipid-SPMNP and DMSA-SPMNP. However, higher concentration of COOH-lipid-SPMNP or PEG-lipid-SPMNP seems to have no improvement in magnetic isolation. Hence, NH_2_-lipid-SPMNP and DMSA-SPMNP were selected to study further SPMNP-cell interaction.

**Fig. 5:**
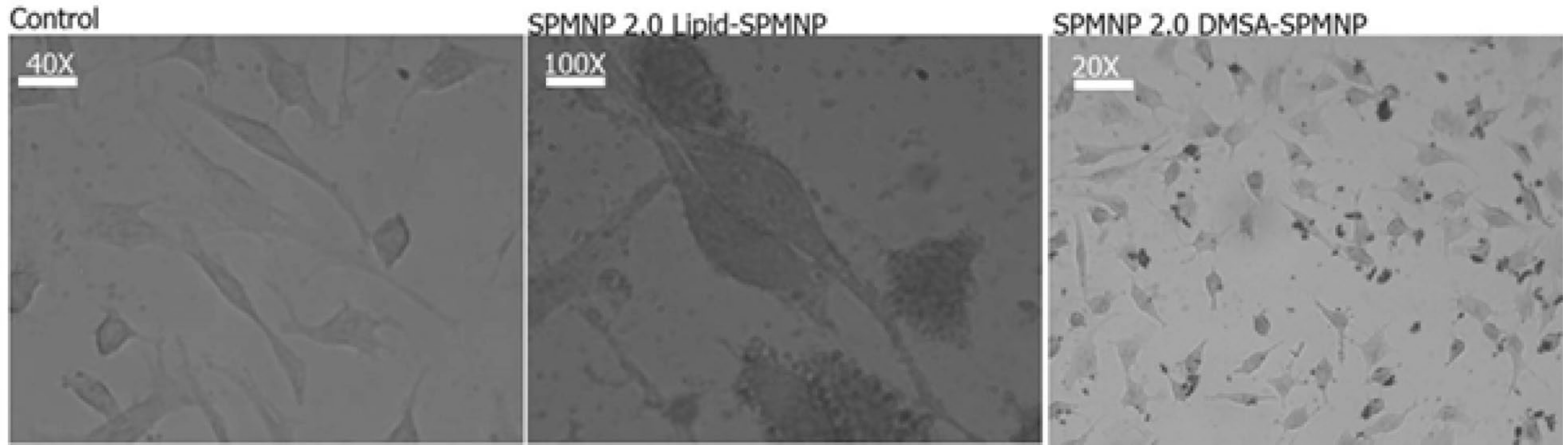
Prussian blue staining for HeLa cells incubated with Lipid-SPMNP and DMSA-SPMNP in DMEM medium for pulse incubation period of 20 min

**Fig. 6:**
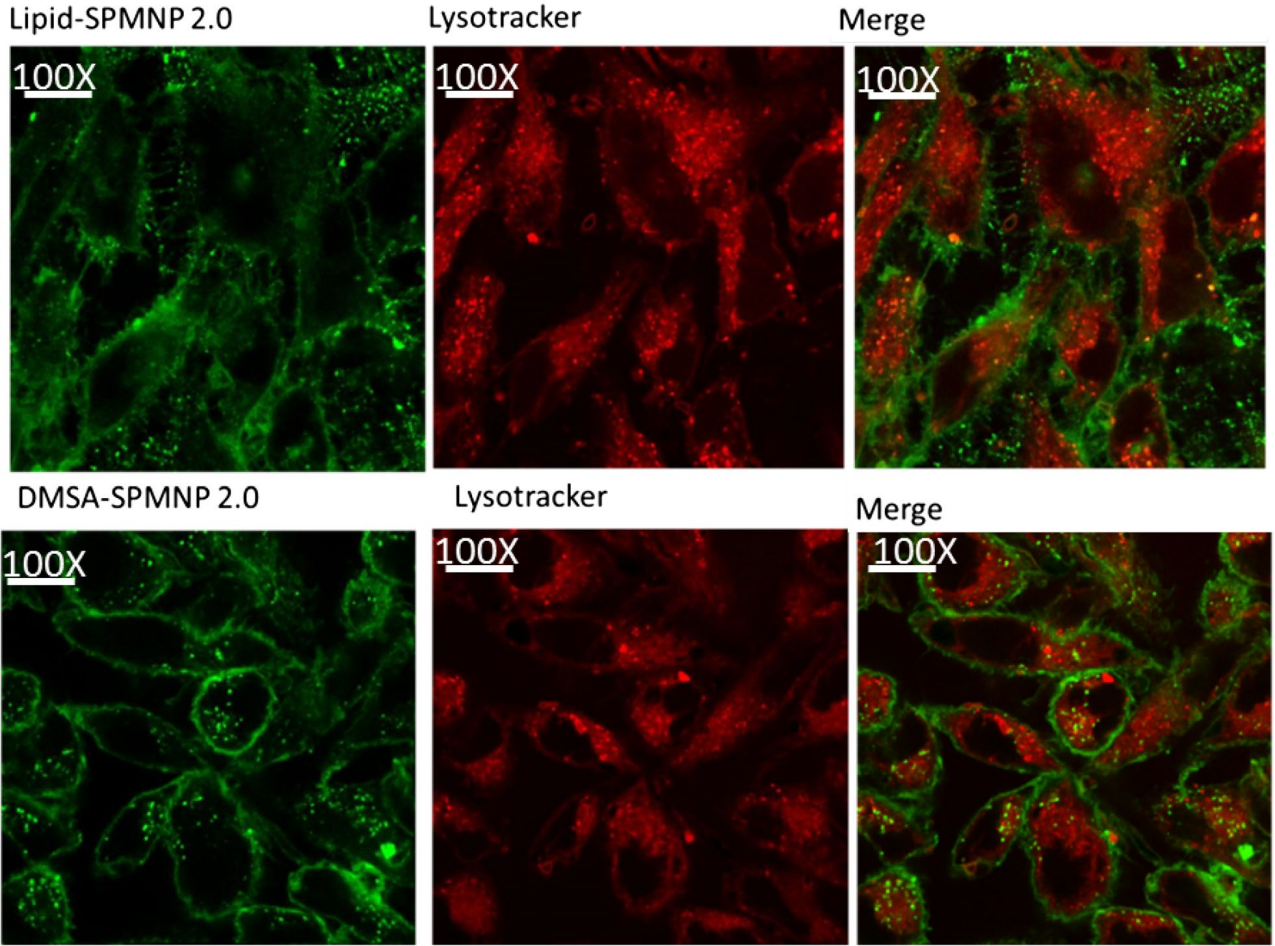
Confocal analysis for pulse 15 min and chase 30 min for HeLa cells incubated with FluorLipid-SPMNP and FluorDMSA-SPMNP and stained with Lysotracker for acidic compartments

Figure 5 shows Prussian blue staining of NH_2_-lipid-SPMNP and DMSA-SPMNP incubated HeLa cells. Using Prussian blue staining, we optimized the pulse incubation period for both NH_2_-lipid-SPMNP and DMSA-SPMNP. We tested SPMNP localization after pulse incubation period for 20 min and observed that majority of the SPMNPs are localized at the cell surface through blue staining. Similarly we confirmed the cell surface localization using Fluorlipid-SPMNP and FluorDMSA-SPMNP in confocal image setting. We performed pulse for 15 min with nanoparticle incubation and chase for 30 min after removing excess nanoparticle. In Fig. 6, we observed that majority of fluorlipid- SPMNP are at the cell surface and FluorDMSA-SPMNP are internalized in the cell. We also co-stained HeLa cells with lysotracker that stains the acidic compartments in the cells. We observed that there is no co-localization of fluorescent SPMNP with lysotracker. In addition we observed fluorescent SPMNP only at the cell surface. Although we observed most of the Fluorlipid-SPMNPs are at cell surface, a population of SPMNP was internalized and was identified to be localized in the cytosolic region of the cell. No fluorlipid-SPMNP was observed to be localized in the nucleus. Similarly, no fluorDMSA was observed to be localized in nucleus.

### Subcellular fractionation

**Fig. 7:**
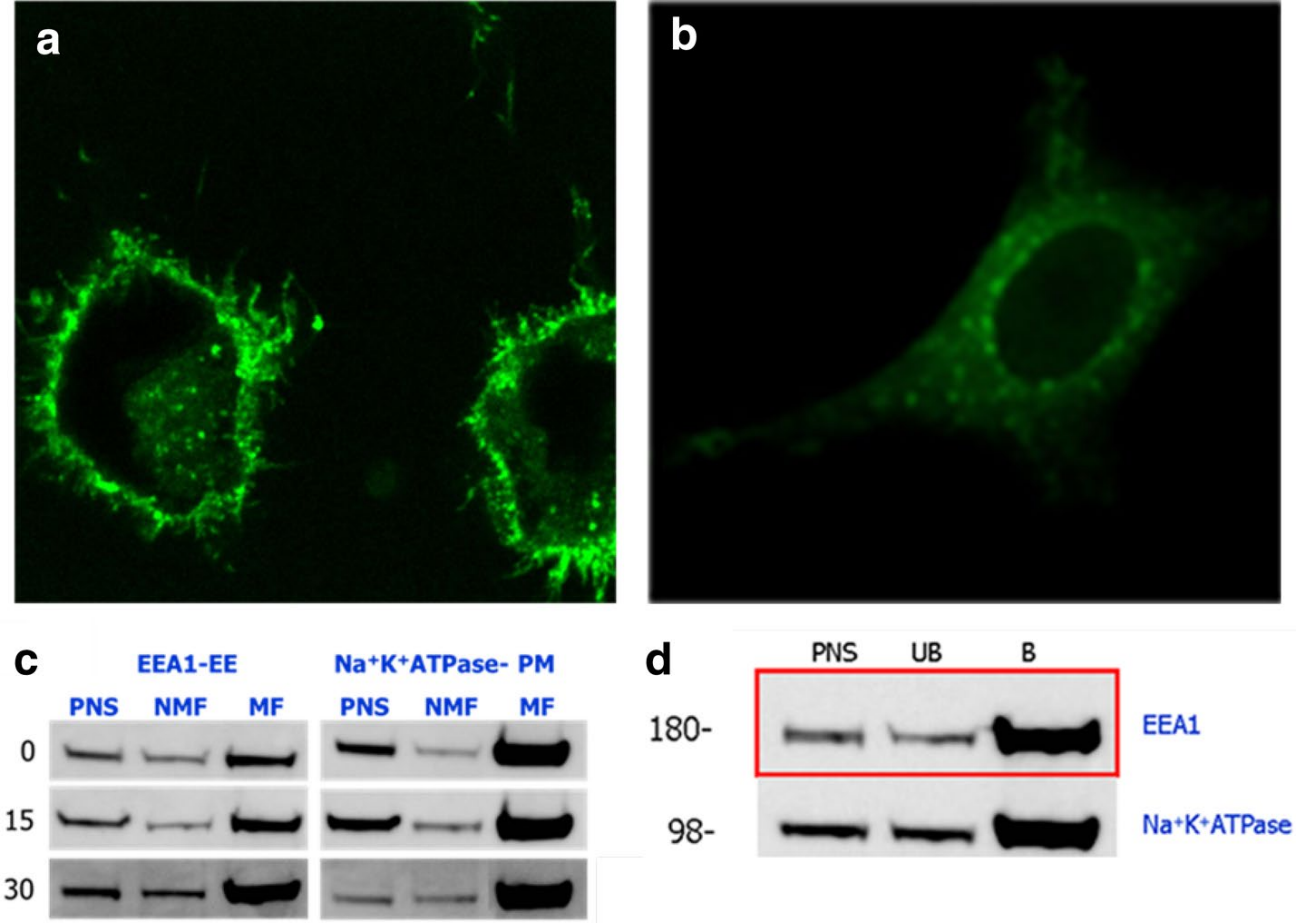
SPMNP based Subcellular fractionation: **a** FluorLipid-SPMNP incubated with HeLa cells for pulse 15 min and chase 240 min; **b** FluorDMSA-SPMNP incubated with HeLa cells for pulse 15 min and chase 240 min; **c** Western blot analysis for EEA1—early endosomes and Na^+^K^+^ATPase—plasma membrane marker for NH_2_-Lipid-SPMNP incubated with HeLa cells for pulse 15 min and chase 0, 15 and 30 min; **d** Western blot analysis for early endosomes and plasma membrane marker for DMSA-SPMNP incubated with HeLa cells for pulse 15 min and chase 240 min. Red Label represents the enrichment of EEA1 which is a marker for early endosomes

By increasing the chase period for 240 min, we found in Fig. 7a, b that Fluorlipid-SPMNP was predominately at the cell surface, while FluorDMSA-SPMNP was localized inside the cells. Both the fluorlipid-SPMNP and FluorDMSA-SPMNP were not internalized into the nucleus. However, preliminary comparison of the percentage of SPMNP at the cell surface and that are internalized into cytoplasm shows that majority of the Fluorlipid-SPMNP are at the cell surface with a subset population internalized. While, majority of the FluorDMSA-SPMNP are internalized in the cytosol, a subset of the population is at the cell surface. Western blot analysis of magnetic subcellular fractions for various pulse–chase paradigm showed that Na^+^K^+^ATPase (Plasma membrane marker) were enriched for NH_2_-lipid-SPMNP pulsed cells for incubation periods of up to 30 min, while EEA1 (early endosome marker) were enriched for DMSA-SPMNP pulsed cells at 240 min incubation with no additional increase in Na^+^K^+^ATPase (Plasma membrane marker) enrichment (Fig. 7c, d). These data show that cell surface localization of NH_2_-lipid-SPMNP, NH_2_-lipid-SPMNP based subcellular fractionation results in Plasma membrane enrichment. Time dependent internalization of DMSA-SPMNP would allow enrichment of subcellular compartment such as early endosomes, late endosome and lysosomes based on the chase period.

## Conclusion

**Fig. 8:**
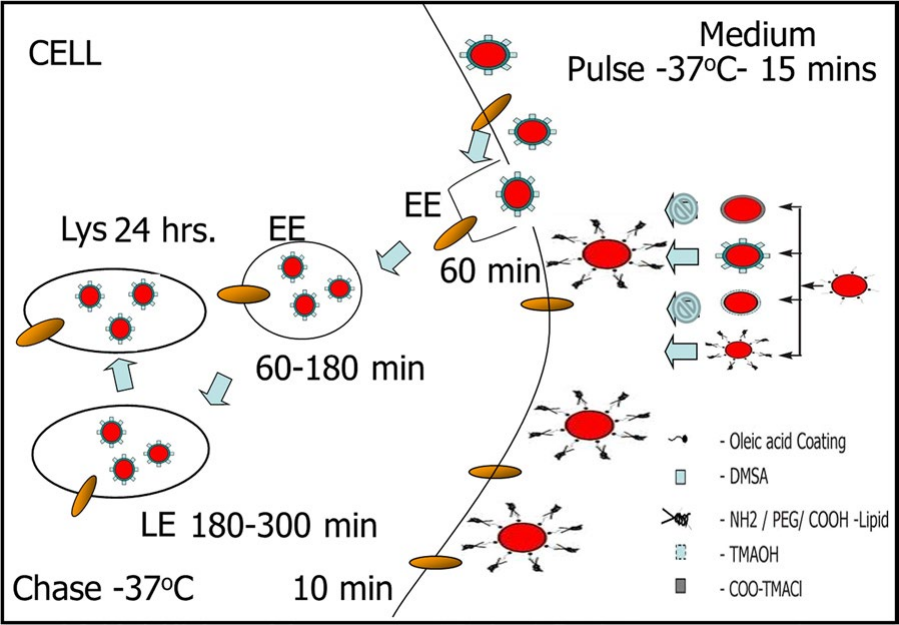
Pulse–chase methodology using SPMNP: schematic representation of our hypothesis on pulse–chase methodology with cell surface localization of lipid-SPMNP and subcellular localization of DMSA-SPMNP as shown previously by [15, 34, 41]

In this article, we show that surface functionalization of SPMNP governs SPMNP-cell interaction such as subcellular localization of DMSA-SPMNP and cell surface localization of lipid-SPMNP. By combining classical cell biology approach of pulse–chase paradigm with magnetic fractionation, we propose a methodology to isolate plasma membrane using NH_2_-lipid-SPMNP, endosomes and lysosomes using DMSA-SPMNP. To isolate highly pure and high yield subcellular compartments, we propose optimal pulse–chase paradigm with 15 min of pulse with NH_2_-lipid-SPMNP at 4 °C or 37 °C. Particularly, the chase period without DMSA-SPMNP governs subcellular compartmental isolation such as 60–180 min for early endosomes, 180–300 min for late endosomes and 300 min to 24 h for lysosomes (Fig. 8). By isolating plasma membrane or lysosomes using SPMNP technology; several research groups have performed subcellular omics and generated comprehensive data on the biomolecular composition of isolated organelles for both native and diseased conditions [11, 15, 41]. Previously, we used phospholipid functionalized SPMNP to isolate cell membrane from Wild-type (wt), Presenilin double knockout (PSENdKO) and human Presenilin-1rescue (hPSEN1rescue) mouse embryo fibroblasts (MEF). Using mass spectrometry on isolated high pure cell membrane fraction, we generated comprehensive cell surface omics for proteins (proteomics), lipids (lipidomics) and glycoproteins (glycomics). By analyzing the biomolecular composition of plasma membrane and total cell lysate, we were able to identify distinct protein and lipid population that were localized predominately at the cell surface (for example: Cholesterol). By analyzing the biomolecular composition in disease condition (PSENdKO), we observed that cholesterol was less abundant at the cell surface compared to the cytosol. We also confirmed that rescue phenotype (hPSEN1rescue) were able to have similar levels of cholesterol at the cell surface as it is in wt. Similar biomolecular localization was observed for several proteins such as Caveolin-1, Myoferlin and β-dystroglycan. By using SPMNP based subcellular fractionation, several groups have isolated endosomes and lysosomes with high purity-yield for subcellular omics. Negatively charged SPMNPs (like Dextran functionalized) were extensively applied for lysosomal fractionation [42]. Using magnetic fractionation, several research groups were able to decipher endosomal trafficking in lysosomal storage disorders (Niemann–Pick type C) [25]. Due to generic nature of SPMNP, our methodology could be used to isolate subcellular compartments from any given adherent cells. In addition, the methodology does not include any acidic treatment or antibody based pulldown and subcellular compartments are isolated under native physiological conditions [43]. Hence, this methodology would facilitate enzymatic studies, isolating intact membrane protein complexes and structural studies. With additional optimization step, our methodology could be applied for suspension cells [44, 45]. As a future perspective, our methodology can be used to isolate subcellular compartments in primary cells and can be extended to in vivo studies.

## References

[1] Wedeking T (2015). Spatiotemporally controlled reorganization of signaling complexes in the plasma membrane of living cells. S mall.

[2] Grecco HE, Schmick M, Bastiaens PI (2011). Signaling from the living plasma membrane. Cell.

[3] Settembre C (2013). Signals from the lysosome: a control centre for cellular clearance and energy metabolism. Nat. Rev. Mol. Cell Biol..

[4] Hu YB (2015). The endosomal–lysosomal system: from acidification and cargo sorting to neurodegeneration. Transl. Neurodegener..

[5] Semple JW, Ellis J, Delovitch TL (1989). Processing and presentation of insulin. II. Evidence for intracellular, plasma membrane-associated and extracellular degradation of human insulin by antigen-presenting B cells. J Immunol.

[6] Lopes L (2006). Phagocytosis, endosomal/lysosomal system and other cellular aspects of macrophage activation by Canova medication. Micron.

[7] Cameron IL (1988). Role of plasma membrane and of cytomatrix in maintenance of intracellular to extracellular ion gradients in chicken erythrocytes. J. Cell. Physiol..

[8] Leth-Larsen R, Lund RR, Ditzel HJ (2010). Plasma membrane proteomics and its application in clinical cancer biomarker discovery. Mol. Cell. Proteomics.

[9] Corte-Real L (2013). Cellular uptake mechanisms of an antitumor ruthenium compound: the endosomal/lysosomal system as a target for anticancer metal-based drugs. Microsc. Microanal..

[10] Pasternak SH, Callahan JW, Mahuran DJ (2004). The role of the endosomal/lysosomal system in amyloid-beta production and the pathophysiology of Alzheimer’s disease: reexamining the spatial paradox from a lysosomal perspective. J. Alzheimers Dis..

[11] Sannerud R (2016). Restricted location of PSEN2/gamma-secretase determines substrate specificity and generates an intracellular abeta pool. Cell.

[12] Bagshaw RD, Mahuran DJ, Callahan JW (2005). Lysosomal membrane proteomics and biogenesis of lysosomes. Mol. Neurobiol..

[13] Stasyk T (2007). Identification of endosomal epidermal growth factor receptor signaling targets by functional organelle proteomics. Mol. Cell. Proteomics.

[14] Yokoyama T (2013). Plasma membrane proteomics identifies bone marrow stromal antigen 2 as a potential therapeutic target in endometrial cancer. Int. J. Cancer.

[15] Thimiri Govinda Raj DB (2011). A novel strategy for the comprehensive analysis of the biomolecular composition of isolated plasma membranes. Mol. Syst. Biol..

[16] Garver WS, Heidenreich RA (2002). The Niemann–Pick C proteins and trafficking of cholesterol through the late endosomal/lysosomal system. Curr. Mol. Med..

[17] Thimiri Govinda Raj DB, Khan NA (2016). Designer nanoparticle: nanobiotechnology tool for cell biology. Nano Converg..

[18] Jones DH, Matus AI (1974). Isolation of synaptic plasma membrane from brain by combined flotation-sedimentation density gradient centrifugation. Biochim. Biophys. Acta.

[19] Zhang W (2003). Affinity enrichment of plasma membrane for proteomics analysis. Electrophoresis.

[20] Guimaraes de Araujo ME, Huber LA, Stasyk T (2011). Latex beads internalization and quantitative proteomics join forces to decipher the endosomal proteome. Expert Rev. Proteomics.

[21] Zhang W (2011). Coating cells with cationic silica-magnetite nanocomposites for rapid purification of integral plasma membrane proteins. Proteomics.

[22] Jain A (2012). Single-molecule pull-down for studying protein interactions. Nat. Protoc..

[23] Orr GA (1981). The use of the 2-iminobiotin-avidin interaction for the selective retrieval of labeled plasma membrane components. J. Biol. Chem..

[24] Raghunath AT (2014). Novel surface functionalized superparamagnetic nanoparticles with engineered cellular uptake as a means for intracellular omics. Mol. Biol. Cell.

[25] Diettrich O (1998). Application of magnetic chromatography to the isolation of lysosomes from fibroblasts of patients with lysosomal storage disorders. FEBS Lett..

[26] Baravalle G (2005). Transferrin recycling and dextran transport to lysosomes is differentially affected by bafilomycin, nocodazole, and low temperature. Cell Tissue Res..

[27] Cordwell SJ, Thingholm TE (2010). Technologies for plasma membrane proteomics. Proteomics.

[28] Nowacek A (2013). Methods for isolation and identification of nanoparticle-containing subcellular compartments. Methods Mol. Biol..

[29] D.B.T.G. Raj, et al., Development and application of SPMNPs for subcellular proteomics, in International Conference for Micro and Nanotechnologies for BioScience (2008)

[30] D.B.T.G. Raj, et al., Cellular uptake of superparamagnetic nanoparticles as a strategy to isolate and characterize endosomal compartments, in Frontiers Annual meeting Leuven, (Leuven, 2007)

[31] Sun S, Zeng H (2002). Size-controlled synthesis of magnetite nanoparticles. J. Am. Chem. Soc..

[32] Dubertret B (2002). In vivo imaging of quantum dots encapsulated in phospholipid micelles. Science.

[33] D.B. Thimiri Govinda Raj, Fluorescent nano-switches for diagnostic application. Protoc. Exch. 2015. 10.1038/protex.2015.125

[34] D.B.T.G. Raj, Superparamagnetic nanoparticle based (10 nm) isolation of plasma membrane for high resolution mass spectrometry analysis of the proteome, lipidome and glycome, in Semiconductor Physics Section. (Leuven, 2011)

[35] Huang X (2007). Self-assembled virus-like particles with magnetic cores. Nano Lett..

[36] J. Trekker, D.B.T.G. Raj, et al., Effect of the core size of monodisperse superparamagnetic nanoparticles on their relaxometric enhancing properties for MRI, in 8th International Conference on Scientific and Clinical Applications of Magnetic Carriers, (Rostock, 2010), p. 52

[37] Lee JH (2007). Artificially engineered magnetic nanoparticles for ultra-sensitive molecular imaging. Nat. Med..

[38] T.G. Deepak Balaji, et al., Biarsenical nanoparticles for in vivo labeling. Patent WO2013076159 (2013)

[39] Brun E, Sicard-Roselli C (2014). Could nanoparticle corona characterization help for biological consequence prediction?. Cancer Nanotechnol.

[40] D.B.T.G. Raj, Surface coating dependent selection of Superparamagnetic Nanoparticles for whole cell isolation, in 8th International Conference on the Scientific and Clinical Applications of Magnetic carrier, (Rostock, 2010)

[41] Tharkeshwar AK (2017). A novel approach to analyze lysosomal dysfunctions through subcellular proteomics and lipidomics: the case of NPC1 deficiency. Sci. Rep..

[42] Becich MJ, Baenziger JU (1991). Ligand-specific isolation of endosomes and lysosomes using superparamagnetic colloidal iron dextran glycoconjugates and high gradient magnetic affinity chromatography. Eur. J. Cell Biol..

[43] Raj DBTG (2012). A novel strategy for the comprehensive analysis of the biomolecular composition of isolated plasma membranes. FEBS J..

[44] D.B. Thimiri Govinda Raj, Protocol for eukaryotic plasma membrane isolation using superparamagnetic nanoparticles. J. Magn. Magn. Mater. 2017. 10.1016/j.jmmm.2017.12.0

[45] D.B. Thimiri Govinda Raj, Synthesis of hybrid AuNPs functionalised superparamagnetic NPs. Micro Nano Lett. 2017. 10.1049/mnl.2017.0574

